# Coexistence of carcinoma and tuberculosis in one breast

**DOI:** 10.1186/1477-7819-6-29

**Published:** 2008-03-04

**Authors:** Ahmed Alzaraa, Neha Dalal

**Affiliations:** 1 Department of General surgery, Tameside General Hospital, Manchester, UK; 2 Department of Histopathology, Tameside General Hospital, Manchester, UK

## Abstract

**Background:**

The coexistence of breast cancer and tuberculosis is very rare. This can create a dilemma in the diagnosis and treatment as there are no pathognomonic symptoms or signs to distinguish both diseases.

**Case presentation:**

A female patient was seen in the breast clinic for a right breast lump. Clinical examination and investigation confirmed cancer and tuberculosis of the right breast. She underwent right mastectomy and axillary clearance and received chemo and radiotherapy. Unfortunately, she died of wide spread metastases.

**Conclusion:**

The simultaneous occurrence of these two major illnesses in the breast can lead to many problems regarding diagnosis and treatment. Though rare, surgeons, pathologists and radiologists should be aware of such condition.

## Background

The coexistence of carcinoma and tuberculosis (TB) of the breast and the axillary lymph nodes is rare. The clinical situations that arise are the presence of carcinoma and tuberculous mastitis, carcinoma in the breast with axillary tuberculous adenitis or both.

## Case presentation

A 47 years old Asian lady was seen in the breast clinic in July 2004 for a rapidly increasing lump in the right breast which had been present for four months. There was no nipple discharge and no family history of breast cancer. He mother in law died of pulmonary tuberculosis about 10 years ago.

Clinical examination revealed a 6 cm × 8 cm mass in the right breast with nipple retraction. There was also a 2 cm × 2 cm palpable lymph node in the right axilla.

**Figure 1:**
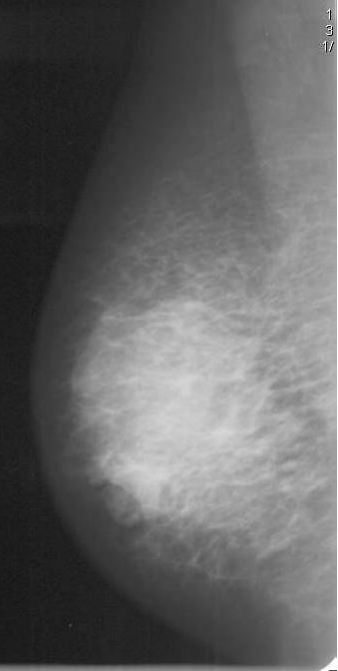
Mammogram of the right breast showing the increased asymmetric density in the right retro-areolar with some skin thickening of the areola and retraction of the nipple.

**Figure 2:**
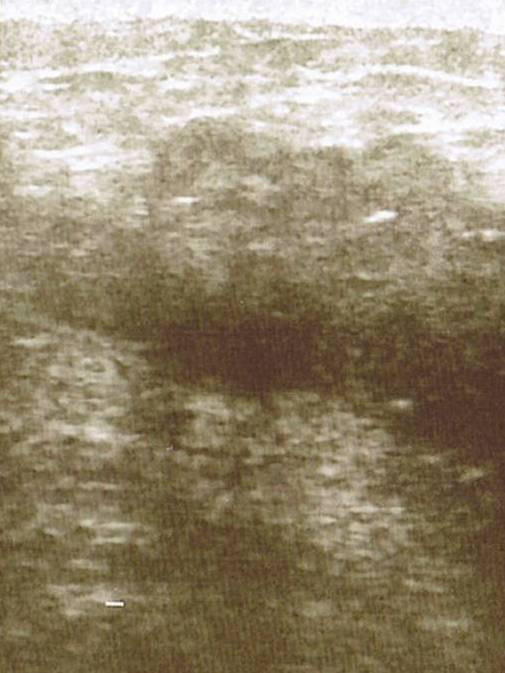
Ultrasound scan of the right breast showing showing the hypodense irregular areas in position close to the areola with some distal shadowing.

**Figure 3:**
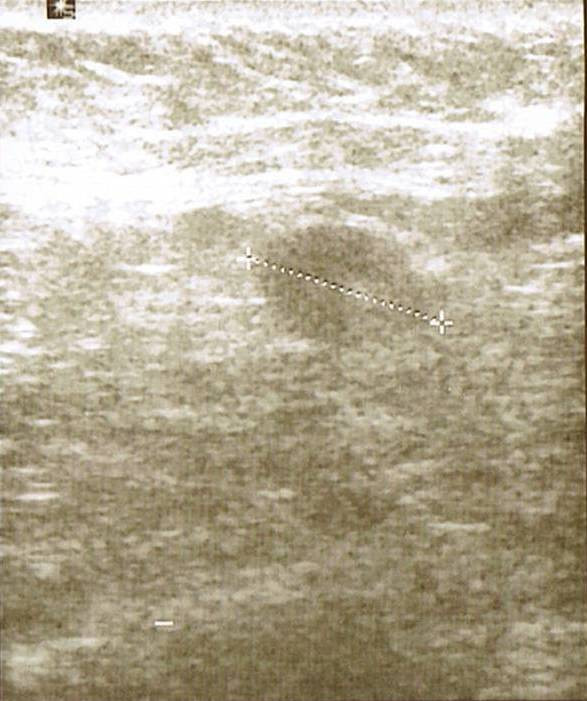
The lymph node is shown on ultrasound with some cortical thickening at its distal pole suggesting focal metastasis.

Mammogram showed asymmetric increased density in the right retro-areolar area with some skin thickening of the areola and some retraction of the nipple (Figure 1). Foci of fine calcification were also noted in both breasts. Ultrasound of the right breast revealed widespread hypodense irregular areas extending from 7–10 O'clock in position close to the areola with some distal shadowing (Figure 2), raising the suspicion of infiltrating ductal carcinoma. There was also a 1.3 cm × 1.9 cm lymph node with some cortical thickening at its distal pole which suggested some focal metastasis (Figure 3).

Fine needle aspiration of the mass was inadequate. A tru-cut biopsy confirmed an invasive ductal carcinoma of no special type along with evidence of non-necrotising granulomatous inflammation containing multinucleated Langhans type giant cells. Subsequent Z-N staining for acid fast bacilli showed multiple bacilli within macrophages, confirming a tuberculous aetiology. Erythrocyte Sedimentation Rate was 25 mm/h. She was commenced on antituberculous treatment.

**Figure 4:**
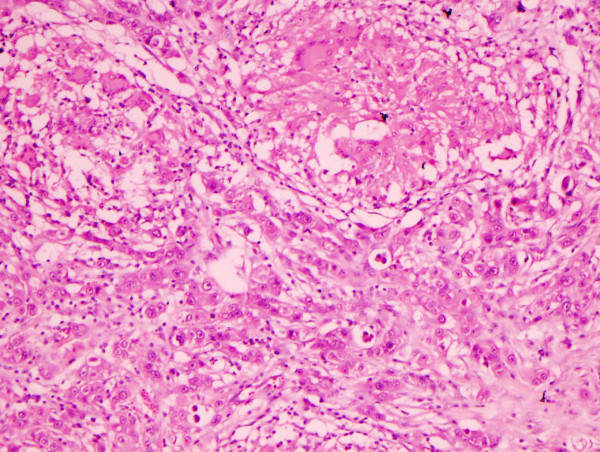
Infiltrating ductal carcinoma in the lower half of the field with two epithelioid granulomata containing multinucleated giant cells in the upper half of the field(H&E 10×).

**Figure 5:**
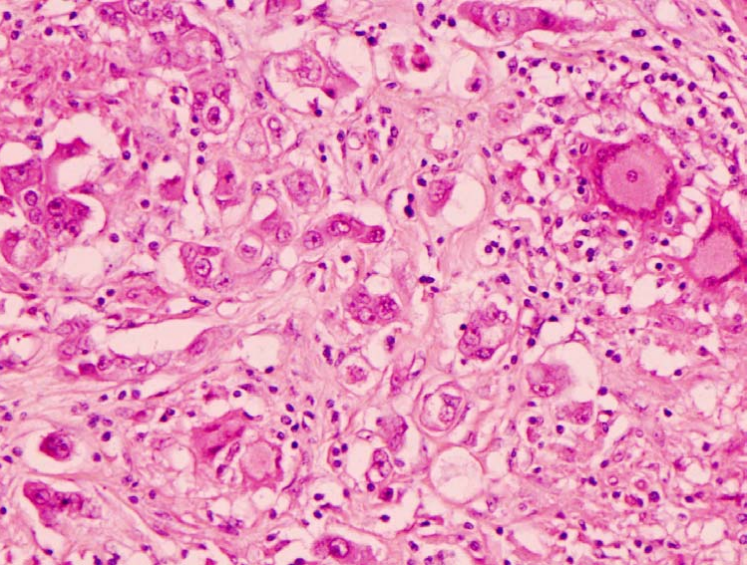
Higher power view of infiltrating ductal carcinoma with an epithelioid granuloma containing Langhan's type giant cells in the upper right hand corner of the field (H&E 20×).

She underwent a right mastectomy with axillary node sampling which showed a 5.5 cm × 5.0 cm × 3.0 cm, grade-II invasive ductal cell carcinoma which was multifocal, with the largest focus measuring 33 mm. Florid lymphovascular invasion was seen along with low grade ductal carcinoma in situ. A striking granulomatous inflammation was seen within the surrounding stroma with multiple non-necrotising epithelioid containing granulomata (Figures 4 &5). Ten of the thirteen indentified lymph nodes showed metastatic carcinoma, and one lymph node showed multiple epithelioid granulomas. TNM classification was pT3, pN3a, pMx. Since the patient had already been commenced on antituberculous treatment prior to surgery, special stains for acid fast bacilli were negative in this specimen.

Chest X-Ray, abdominal ultrasound, small bowel follow through and isotope bone scan were normal. The patient received adjuvant eight courses of FEC (Fluorouracil, Epirubicin and Cyclophosphamide), and a course of radiotherapy to the right chest wall, supraclavicular fossa and axilla (40 Gy in 15 Fractions). The right chest wall was fitted with 8 MeV electrons, and the supraclavicular foaas and axilaa were fitted with 8 MeV photons. Subsequently, she had wide spread metastases with pleural and pericardial effusion which were drained. She was commenced on weekly Paclitaxel with three weekly Herceptin. Unfortunately, she died in April 2007 before finishing the treatment.

## Discussion

Granulomatous inflammation of the breast is an inflammatory process with multiple aetiologies. It can be caused by breast cancer, tuberculosis, granulomatous mastitis (GM), sarcoidosis, fungal infections such as actinomycosis, parasites such as filariasis, Wegener's granulomatosis, duct ectasia, brucellosis and traumatic fat necrosis [1]. GM has characteristic histological features, the most important of which is predominantly lobular inflammatory disease, hence the term Granulomatous Lobular Mastitis (GLM) [2]. Most patients with GM present with a well-defined hard breast lump which may be associated with diffuse nodularity, nipple retraction, skin fistulas, fixation to skin or underlying tissues [3,4,1]. The cytomorphologic pattern seen in tuberculous mastitis (TM) is indistinguishable from that seen in GLM. Since it is not always possible detect acid – fast bacilli in histologic sections of TM, accurate diagnosis can safely be made only when additional clinical data is present [1]. The coexistence of carcinoma and tuberculosis (TB) of the breast and the axillary lymph nodes is rare and was first reported by Pilliet and Piatot in 1897 [5-7]. TM is rare even in countries where tuberculosis is still common, accounting for only 0.1% of all cases [5,8]. This is probably due to increased breast tissue resistance to the survival and multiplication of Mycobacterium bacilli, antituberculous treatment, and underdiagnosis of TM [8]. Hani-Bani K, et al [8] believed that immigration from endemic areas, and the increasing prevalence of immunosuppressive disorders, including HIV infection, might be responsible for increasing the incidence of TM in Western countries in the future. Therefore, a high index of suspicion might be justified in immigrants from regions with a high prevalence of tuberculosis, for example, or atypical clinical or radiological presentations. The breast can be involved by a penetrating wound of the skin of the breast; the lactiferous ducts via the nipple; direct extension from the lungs and the chest wall; the blood stream and the lymphatics [6]. It is generally believed that tuberculous infection of the breast is usually secondary to a pre-existing tuberculous focus located elsewhere in the body. Such a pre-existing focus could be of pulmonary origin or could be a lymph node within the paratracheal, internal mammary, or axillary nodal basin [9]. Histologically, TM can be classified into nodular which mimics carcinoma; disseminated which causes caseation and sinus formation; and sclerosing which grows slowly with no suppuration [8].

The clinical situations that arise are the presence of carcinoma and tuberculous mastitis, carcinoma in the breast with axillary tuberculous adenitis or both [6]. There does not appear to be a casual link between mammary tuberculosis and breast cancer, and there is no evidence that TB is carcinogenic at any site [10]. The simultaneous occurrence of carcinoma and tuberculosis can lead to many problems regarding diagnosis and treatment as there are no pathognomonic symptoms or signs to distinguish breast tuberculosis from breast cancer, especially if the upper outer quadrant is involved [6-8]. An isolated breast mass without an associated sinus tract can commonly mimic the presentation of breast cancer, since the clinically palpable breast mass is usually firm, ill-defined, irregular, and can be associated with fixation to the skin [9]. The radiological features of TM are non-specific, mimicking those of many diseases including breast cancer. Ultrasound scan usually reveals homogenous, irregular hypoechoic lesions with focal posterior shadowing, or multiple circumscribed heterogenous hypoechoic lesions associated with a large mass [4]. A unique finding strongly suggestive of TM is the presence of a dense sinus tract connecting an ill-defined breast mass to localised skin thickening and bulge [8]. Most decisions in the management of breast cancer are taken based on TNM staging of the tumours. This can lead to overestimation of the tumour size, therefore, these patients lose the opportunity for breast conservation due to this [6]. The key to proper treatment is biopsy of the lesion [7]. If breast cancer is clinically operable, radical mastectomy is indicated, followed by postoperative antituberculous chemotherapy for 18 months, and if the cancer is incurable, palliative measures combined with antituberculous drugs are indicated [7].

## Conclusion

The existence of tuberculosis and carcinoma in the breast is very rare. Their clinical and radiological presentations are very similar. Histology remains the keystone in confirming the diagnosis. Full liaison between surgeons, radiologists and pathologists is very important to plan best management of such conditions.

## Competing interests

The author(s) declare that they have no competing interests.

## Authors' contributions

AA: Performed literature review, drafted and revised manuscript. ND: Evaluated histopathological features.

## References

[B1] Akan A, Akyildiz H, Deneme M, Akgun H, Aritas Y (2006). Granulomatous lobula mastitis: a complex diagnostic and therapeutic problem. World J Surg.

[B2] Going J, Anderson T, Wilkinson S, Chetty U (1987). Granulomatous lobular mastitis. J Clin Pathol.

[B3] Heer R, Shrimankar J, Griffith C (2003). Granulomatous mastitis can mimic breast cancer on clinical, radiological or cytological examination: a cautionary tale. The Breast.

[B4] Tuncbilek N, Karakas H, Okten O (2004). Imaging of granulomatous mastitis: assessment of three cases. The Breast.

[B5] Ballini A, Zaritzky A, Lupo L (1989). Breast tuberculosis and carcinoma. Isr med sci.

[B6] Tulasi N, Raju P, Damodaran V, Radhika T (2006). A spectrum of coexistent tuberculosis and carcinoma in the breast and axillary lymph nodes: Report of five cases. The breast.

[B7] Miller R, Salomon P, West J (1971). The coexistence of carcinoma and tuberculosis of the breast and axillary lymph nodes. Am J Surgery.

[B8] Bani-Hani K, Yaghan R, Matalka I, Mazahreh T (2005). Tuberculous mastitis: a disease not to be forgotten. Int J tuberc Lung Dis.

[B9] Akcay M, Saglam L, Polat P, Erdogan F, Albayrak Y, Povoski S (2007). Mammary tuberculosis-importance of recognition and differentiation from that of a breast malignancy: report of three cases and review of the literature. World J Surg Oncol.

[B10] Robinson A, Horne C, Weaver A (2001). Coexistence of axillary tuberculous lymphadenitis with lymph node metastases from a breast carcinoma. Clin Oncol.

